# Structural dynamics of a methionine *γ*-lyase for calicheamicin biosynthesis: Rotation of the conserved tyrosine stacking with pyridoxal phosphate

**DOI:** 10.1063/1.4948539

**Published:** 2016-04-29

**Authors:** Hongnan Cao, Kemin Tan, Fengbin Wang, Lance Bigelow, Ragothaman M. Yennamalli, Robert Jedrzejczak, Gyorgy Babnigg, Craig A. Bingman, Andrzej Joachimiak, Madan K. Kharel, Shanteri Singh, Jon S. Thorson, George N. Phillips

**Affiliations:** 1 Biosciences at Rice, Rice University, 6100 Main St., Houston, Texas 77005, USA; 2 Biosciences Division, Midwest Center for Structural Genomics, Argonne National Laboratory, Bldg. 446/Rm. A104, 970 South Cass Avenue, Argonne, Illinois 60439, USA; 3 Department of Biochemistry, University of Wisconsin-Madison, Madison, Wisconsin 53706, USA; 4 Department of Pharmaceutical Sciences, College of Pharmacy, University of Kentucky, Lexington, Kentucky 40536, USA

## Abstract

CalE6 from *Micromonospora echinospora* is a (pyridoxal 5′ phosphate) PLP-dependent methionine *γ*-lyase involved in the biosynthesis of calicheamicins. We report the crystal structure of a CalE6 2-(N-morpholino)ethanesulfonic acid complex showing ligand-induced rotation of Tyr100, which stacks with PLP, resembling the corresponding tyrosine rotation of true catalytic intermediates of CalE6 homologs. Elastic network modeling and crystallographic ensemble refinement reveal mobility of the N-terminal loop, which involves both tetrameric assembly and PLP binding. Modeling and comparative structural analysis of PLP-dependent enzymes involved in Cys/Met metabolism shine light on the functional implications of the intrinsic dynamic properties of CalE6 in catalysis and holoenzyme maturation.

## I. INTRODUCTION

Structural dynamics on various time and length scales are inherent properties of biological macromolecules and are often related to their functions. For example, enzymes, which are chosen by nature to lower the energy barrier of transition state conformations, ultimately require finite structural flexibility to advance the catalytic cycle and avoid being trapped in either substrate bound or product bound states. Protein dynamics can be probed not only by solution approaches like nucleic magnetic resonance (NMR) and other spectroscopies but also by a combination of X-ray crystallography and computational modeling.^1,2^ Recent advances in time-resolved serial femtosecond crystallography (TR-SFX) using pulsed ultra-bright X-ray free electron lasers (XFELs) show promise in capturing photoactive proteins in action at atomic level by taking data within a very short time courses (in the order of 100 fs) during which light-induced conformational changes have been previously initiated.^3,4^ Among many computational structural biology methods, molecular dynamics (MD) simulation, elastic network models (ENMs), and ensemble refinement offer ways complementary to experiments to identify motions that occur at a wide range of temporal and spatial scale relevant to biomolecule function. Unlike MD simulation, which yields a time course of conformational trajectory, ENM performs low-frequency normal mode analysis (NMA) based on an harmonic approximation around the starting reference model, which is assumed as a minimum energy conformation.^5–7^ Ensemble refinement describes protein dynamics by fitting ensemble models to diffraction data which accounts for both anisotropic and anharmonic distributions.^8–10^ We have applied ENM and ensemble refinement approaches to extract the dynamic information from the experimental structure of CalE6, a PLP (pyridoxal 5′ phosphate)-dependent methionine *γ*-lyase from *Micromonospora echinospora*, which is encoded by the calicheamicin biosynthetic gene cluster.^11^

**FIG. 1.**
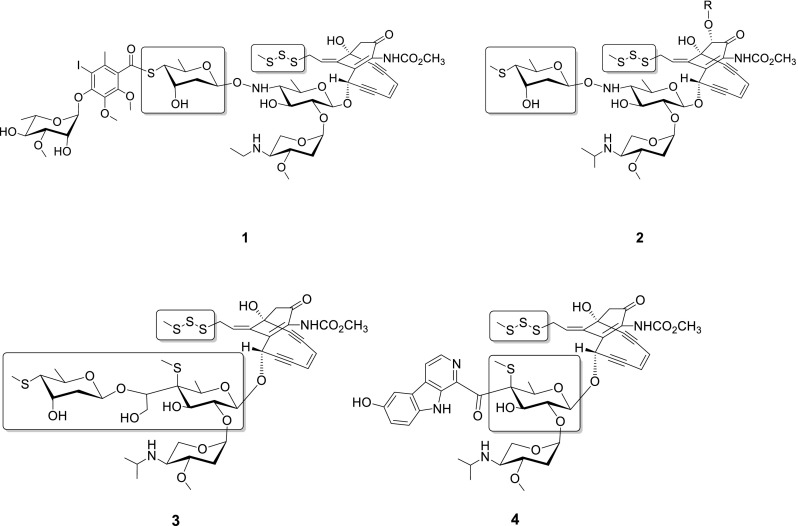
The utilization of sulfur in 10-membered enediynes. The sulfur-containing substructures within calicheamicin (1), esperamicin (2), namenamicin (3), and shishijimicin (4) are highlighted within the boxed areas.

Calicheamicin γ_1_^I^ (Figure 1) is a prototype of the 10-membered enediyne family of antibiotics that contains a characteristic bicyclo[7.3.1]tridecadiynene core.^12–15^ Like all 9- and 10-membered enediynes, calicheamicin-induced oxidative DNA strand scission is enabled by cycloaromatization of the enediyne core to form a highly reactive diradical species.^15,16^ In calicheamicin, this cycloaromatization event is initiated via reductive activation of a unique allylic trisulfide “trigger” and the corresponding reactive diradical is exquisitely positioned by the calicheamicin aryltetrasaccharide, which contains multiple distinctly functionalized sugars that contribute to DNA minor groove recognition and affinity.^15–20^ Given its incredible potency, calicheamicin also served as the warhead of the very first clinically approved monoclonal antibody (mAb) drug conjugate for targeted cancer therapies.^21,22^ Despite that various enzymes involved in biosynthesis of the enedyine core,^22–27^ glycosyltransferation^28,29^ and sugar modification^30–32^ have been biochemically or structurally characterized, the putative enzymes responsible for sulfur mobilization and installation during the formation of enediyne trisulfide and thiosugar (boxed in Figure 1) remain unknown. Protein BLAST-based functional annotation indicates that the calicheamicin biosynthetic cluster contains at least three candidate genes that may encode for enzymes involved in the requisite sulfur biochemistry [*calE4* (putative cysteine desulfurase), *calS4* (putative selenocysteine lyase/cysteine desulfurase), and *calE6* (methionine *γ*- lyase)].^23^ It cannot be ruled out that participation of sulfur-transfer genes from primary-metabolism as reported for the biosynthesis of 2-thiosugar-containing natural product BE-7585A^33^ also occurs during biosynthesis of the 4-thiosugar moiety of calicheamicin. Among the putative genes responsible for sulfur transfer during calicheamicin biosynthesis, the corresponding gene product CalE6 was recently confirmed to show methionine *γ* lyase activity.^34^ However, the contribution of this enzymatic activity to calicheamicin thiosugar and/or trisulfide formation remains unknown.

CalE6 structure was first determined in the 2-(N-morpholino)ethanesulfonic acid (MES) bound form by our group as part of the Protein Structure Initiative (PDB code: 4Q31),^35^ which showed the same homotetrameric overall structure and space group as the phosphate complex structure subsequently reported by Song *et al.* (PDB code: 4U1T).^34^ Despite little structural difference between the two ligand bound forms, all four subunits consistently showed nontrivial local conformational changes involving ∼17° side-chain rotation of the active site residue Tyr 100, a highly conserved residue structurally stacking with the pyridine ring of the cofactor in PLP-dependent enzymes. We further simulated the dynamics and sampled the conformational space of this enzyme using both coarse-grain elastic network models and ensemble refinement methods based on the CalE6-MES complex structure. We demonstrated that computational analysis, complementary to X-ray crystallography not only consistently identified the global distribution of protein mobility but also allowed deriving multiple-conformer ensemble models from diffraction data to properly represent the local dynamics including the rotation mode of active site Tyr 100 of CalE6, as probed by the latter method.

## II. MATERIALS AND METHODS

### A. Cloning and protein expression

Cloning and expression protocols of CalE6 followed the standard high throughput procedures of Midwest Center for Structural Genomics, the details of which are also available at TargetTrack database (www.sbkb.org/tt/) under Project Target ID APC109014, as a Protein Structural Initiative target. A brief summary was provided here. The full length *calE6* gene from *M. echinospora* (gi: 22255867) was amplified from the genomic DNA using forward primer 5′-TACTTCCAATCCAATGCCGTGAGCGGTATGCGCTTCGAC-3′ and reverse primer 5′-TTATCCACTTCCAATGTTAGGTGCCGCCCGCCAG-3′. The PCR product was cloned into vector pMCSG73 according to the ligation-independent procedure and transformed into the *E. coli* BL21(DE3)-Gold strain (Stratagene). The vector pMCSG73 is derived from vector pMCSG53 and contains tRNA genes covering rare codons Arg (AGG/AGA) and Ile (AUA).^36^ DNA sequencing identified a mutation corresponding to D7G variation at the N-terminus, which was subsequently confirmed to show no effect on the overall or remote active site structure of the protein. Supplementary Figure S1 shows the local loss of a salt bridge with Arg 253 from a neighbor subunit and concomitant side-chain reorientation of Arg 253 due to D7G variation without affecting the overall structure.^61^ Selenomethionine labeled protein was overexpressed from *E. coli* BL21 (DE3) culture grown in M9 minimal media supplemented with inhibitory amino acid cocktail and Se-Met under induction condition of 0.5 mM IPTG at 18 °C overnight. The fusion product contains a TVMV-cleavable N-terminal NusA tag followed by a TEV-cleavable N-terminal 6xHis tag (NusA-ETVRFQ/S-HHHHHH-WSHPQFEK-ENLYFQ/SNA-TARGET). After cell lysis by sonication, the Se-Met labeled protein was purified by Ni-NTA affinity chromatography using AKTAxpress system (GE Health Life Sciences, USA), TEV protease cleavage, followed by an additional subtractive IMAC step to remove the protease, uncut protein, and affinity tag. The purified untagged protein was concentrated to 11 mg/ml with Amicon Ultra-15 centrifugal concentrators (Millipore, Bedford, MA, USA) and stored in 20 mM HEPES pH 8.0, 250 mM NaCl, 2 mM dithiothreitol, and 1 mM PLP at −80 °C.

### B. Protein crystallization

Several commercially available crystallization screens (MCSG-1–4, Microlytic, Inc. MA, USA)^37^ were used, which led to identification of multiple CalE6 crystallization conditions. The best diffracting CalE6 crystal was obtained using the sitting drop vapor diffusion method by mixing 0.4 *μ*l of 11 mg/ml protein solution containing 1 mM PLP with 0.4 *μ*l of reservoir solution containing MCSG-2 screen B1 condition (12% w/v PEG 20000 and 0.1 M MES, pH 6.5) using the mosquito liquid dispenser (TTP Labtech, Cambridge, MA, USA). The crystallization drop was equilibrated against a reservoir solution of 140 *μ*l. Crystals were grown at 289 K for 2 weeks, cryoprotected with 25% glycerol and flash-frozen in liquid N_2_.

### C. Data collection and refinement

A set of single-wavelength anomalous diffraction (SAD) data was collected to 2.10 Å resolution at Argonne National Laboratory on the SBC-CAT (19-ID) beamline with the program SBCcollect^38^ using a wavelength 0.97915 Å and an ADSC QUANTUM 315r CCD detector. The dataset was integrated and scaled with HKL3000.^39^ Selenium sites were located using SHELXD^40^ and used for phasing with MLPHARE.^41^ Following density modification,^42^ a partial model was built by three cycles of ARP/wARP.^43^ The structures were completed with alternating rounds of manual model building with COOT^44^ and refinement with PHENIX 1.8.2_1309.^45^ Data collection and refinement statistics were summarized in Table I. All the images of protein models were generated with PyMOL.^46^ The structure factors and refined model were deposited in Protein Data Bank (PDB, www.rcsb.org) as entry 4Q31.^35^ Protein quaternary assembly and buried surface were calculated by PISA server.^47^ Model quality was assessed using MolProbity.^48^ Visualization of 3D structures with PyMOL and COOT was facilitated by stereoscopic HDTV.^49^

### D. Elastic network model

The elastic network model (ENM) of CalE6 was performed using the ElNemo server.^7^ The reference state was set as homotetramer of CalE6 (chains A–D) of crystal structure (PDB code: 4Q31) with removal of water and non-covalent bound molecules, replacement of selenomethionine Se atoms with methionine S, and change of all HETATM records to ATOM. Four PLP cofactors covalently bound to Lys were kept the same as residues LLP 197 in the original PDB model. Default parameters were used from the server. Top five slowest normal modes were analyzed.

### E. Ensemble refinement

The ensemble refinement of CalE6 was run on PHENIX dev_1839.^10,45^ Each subunit of the homotetramer was assigned as a single TLS group. While the extent of ensemble dispersion depends largely on the crystallographic data, global harmonic restraints were applied with a weak weight of 10^−5^ to penalize aberrant movement of the protein segments in the poor electron density regions only beyond a default distance threshold value of 1 Å. Three empirical parameters were optimized during ensemble refinement, where wxray_coupled_tbath_offset = 5.0, ptls = 0.8, and tx = 0.5. The output model number of the ensemble was defined to be 10 to both allow reasonable sampling of the conformations and avoid overfitting to the diffraction data. The refined ensemble models were deposited in PDB as entry 4XQ2. Ensemble refinement moderately improved both *R*_cryst_ and *R*_free_ statistics (Table I).

### F. Dynamics analysis of ensemble models

The ensemble models were analyzed with Mobi server^50^ to extract relative dynamics properties of each residue represented by “average scaled distance” between the same C*α* atoms in all the superposed models. The larger the average scaled distance, the higher relative mobility of the residue in the ensemble models.

## III. RESULTS AND DISCUSSION

### A. The crystal structure of CalE6 and ligand induced rotation of Tyr 100

**FIG. 2.**
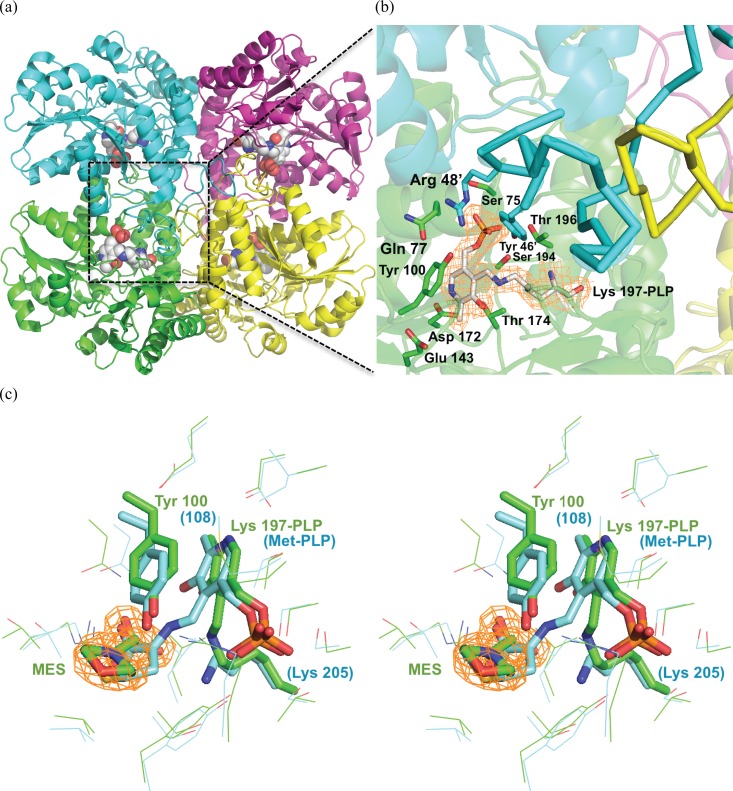
CalE6 overall structure and the active site. (a) Tetrameric structure of CalE6 holoenzyme. Four subunit chains were shown as secondary structure cartoons A (green), B (cyan), C (magenta), and D (yellow). PLP was shown as spheres, with carbon in white, oxygen red, nitrogen blue, phosphorus orange. (b) Active site residues within 4 Å from PLP shown as sticks. 2Fo-Fc electron density map contoured 1.8 Å around PLP was shown at 1.0 σ level. The N-terminal extended loops (residues 14–51) were highlighted as thick ribbons for chains B and D. (c) Stereo images of active site structures of CalE6 (cyan) and *E. histolytica* methionine *γ*-lyase 1 (green) (PDB entry 3AEM).^51^ PLP aldimine complexes and adjacent tyrosine residues were shown as sticks. 2Fo-Fc density map was shown for MES at 1.0 σ level. Structures were aligned using PyMOL^46^ based on the tetrameric form (chains A–D).

The crystal structure of CalE6 was determined and refined to a resolution of 2.1 Å. Two homotetramers were found in the asymmetric unit displaying essentially the same conformational state with overall C*α* RMSD of 0.143 over 1509 residues of four chains (A–D vs. E–F). The buried surface area of individual tetramer is 24,505 ± 325 Å^2^. Each tetramer harbors four molecules of PLP, each bound in a cleft formed at C-2 symmetry-related dimer interfaces (Figure 2(a)). Within the dimer, one subunit provides Lys 197 as the Schiff-base anchor for PLP and forms multiple non-covalent interactions with the cofactor from residues Ser 75, Gln 77, Tyr 100, Glu 143, Asp 172, Thr 174, Ser 194, Thr 196 (Figure 2(b)). The adjacent subunit provides additional charge-charge and hydrogen-bonding interactions with the phosphate group of PLP from residues Arg 48′ and Tyr 46′ located on an extended loop (residues 14–51). The same loop also forms quaternary interactions with symmetry-related loops from a distant subunit to hold together the tetramer (Figure 2(b)). A solvent molecule 2-(N-morpholino)ethanesulfonic acid (MES) was identified in each putative substrate site based on clear electron density (Figure 2(c)), with the sulfonic group of MES located at an equivalent position to the *α*-carboxylic group of an L-Met aldimine catalytic intermediate reported for a homologous methionine *γ*-lyase 1 from *Entamoeba histolytica* (PDB entry 3AEM) (Figure 2(c)).^51^

**FIG. 3.**
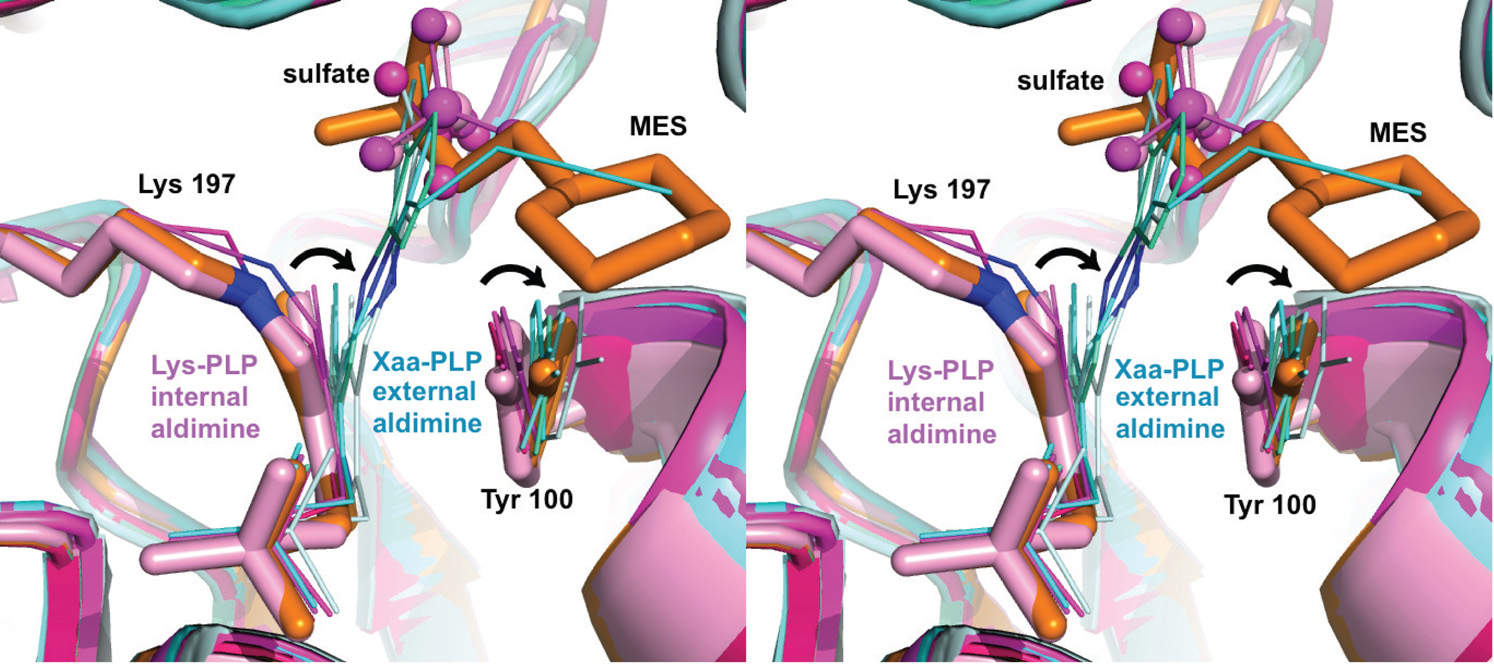
Stereo drawing showing a structural comparison of CalE6 complexes with homologous complexes. Structures were aligned using PyMOL^46^ based on chain A. CalE6 active site residues Tyr 100 and Lys 197-PLP were shown as sticks, in the MES (orange) or sulfate (light pink) bound forms. The corresponding residues of homologs in both internal aldimine (magenta group) and external aldimine (cyan group) forms were shown as lines. MES were shown as sticks, and sulfate ions were shown as spheres. Aldimine linkage nitrogen atoms were colored blue. Similar rotation modes of conformational change of tyrosine and PLP (referring to different forms of an individual enzyme member) were indicated as black arrows. Substrate mimic MES but not small sulfate ion is able to induce rotation of Tyr 100 of CalE6 independent of external aldimine formation and PLP rotation. Concerted rotation motions of the conserved Tyr PLP pair of the homologous structures were observed from internal aldimine to external aldimine transition. Structures aligned include CalE6 (PDB entries 4Q31 and 4U1T),^34,35^ *E. histolytica* methionine *γ*-lyase 1 (PDB entries 3AEM and 3ACZ),^51,52^ *X. oryzae* cystathionine *γ*-lyase (PDB entries 4IY7 and 4IXZ),^53^ and *C. freundii* methionine *γ*-lyase (PDB entries 4HF8, 4OMA, 2RFV).^54–56^ Xaa represents not a particular amino acid.

Despite overall structural similarity between the current CalE6-MES complex and the recently reported CalE6-phosphate complex (PDB 4U1T),^34^ we consistently found a distinct conformational change of Tyr 100 between the two structures for all the subunits of the two tetramers in the asymmetric unit. The major movement of Tyr 100 involves rotation around C*β*-C*γ* bond with minor shift of backbone positions resulting in a 16.5° decrease of C*α*-C*β*-C*γ*-C*δ*1 dihedral angle from 67.4 ± 0.7° to 50.9 ± 2.4° (Figure 3) (Table II). Similar ligand induced conformational changes of the PLP-stacking tyrosine residues were observed for external aldimine reaction intermediates of structural homologs including *E. histolytica* methionine *γ*-lyase 1 (PDB entries 3AEM and 3ACZ, 34% sequence identity with CalE6),^51,52^ *Xanthomonas oryzae* cystathionine *γ*-lyase (PDB entries 4IY7 and 4IXZ, 45% identity),^53^ and *Citrobacter freundii* methionine *γ*-lyase (PDB entries 4HF8, 4OMA, 2RFV, 39% identity) (Figure 3).^54–56^ In all the cases, only the substrates/structural mimics were able to induce the corresponding Tyr side-chain rotation, but not the small anions like sulfate or bicarbonate, which occupy the equivalent positions of the *α*-carboxylic groups of the aldimine intermediate. The C*α*-C*β*-C*γ*-C*δ*1 (χ_2_) dihedral angles of the corresponding PLP-stacking tyrosine residues in these structures were summarized in Table II. The tyrosine side-chain χ_2_ dihedral angles of CalE6 structural homologs analyzed here all fall into statistically allowed but less favored region based on side-chain angle distribution in rotamer libraries derived from high resolution protein structures in Protein Data Bank.^60^ The ligand induced tyrosine χ_2_ angles of CalE6 homologs (49°–59°) deviate further away from the most populated range of around 85°–90° than the corresponding native angles (67°–73°).^60^ These dihedral angle statistics potentially suggest a physically “tensed” or higher energy state of the tyrosine near PLP upon ligand binding compared to the native state. Despite the variation in the ligand-induced torsional angle of the tyrosine, it appears that the proper *π-π* stacking interactions between the tyrosine and PLP were mostly maintained in the external aldimine intermediates as seen for the case of *E. histolytica* methionine *γ*-lyase 1 (Figure 3(c)).^51,52^ Ngo *et al.* have proposed that the catalytic cycle of PLP-dependent enzymes may actively involve PLP conformational changes upon formation of external aldimine as observed in *Xanthomonas oryzae* cystathionine *γ*-lyase.^53^ This PLP conformational change was not observed in the CalE6-MES complex likely because the substrate mimic, lacking the *α*-amino group, is unable to form the external aldimine linkage with PLP.

It is widely accepted that PLP stabilizes the C*α* anion intermediate through extended *π* system delocalization and facilitates catalysis following the formation of the Schiff base linkage with the substrate.^57,58^ The current comparative structural analysis further supports the common mechanism of PLP-dependent enzymes by providing dynamics insights that substrate binding not only induces PLP conformational changes but also causes the concomitant rotation of tyrosine residue that maintains proper *π-π* stacking interactions with the conjugated system extended by the Schiff base linkage. We propose that this concerted motion of PLP and tyrosine pair in response to external aldimine formation significantly contributes to catalysis. The prevalence of this proposed mechanism in PLP-dependent enzymes, if confirmed experimentally, is expected to provide specificity control on the dynamic level in addition to extensively studied stereoelectronic effects, protonation state of the external aldimine intermediates, and specific protein carbanion interactions, which together determine the catalytic outcome.^57,58^

### B. Structural dynamics of CalE6

**FIG. 4.**
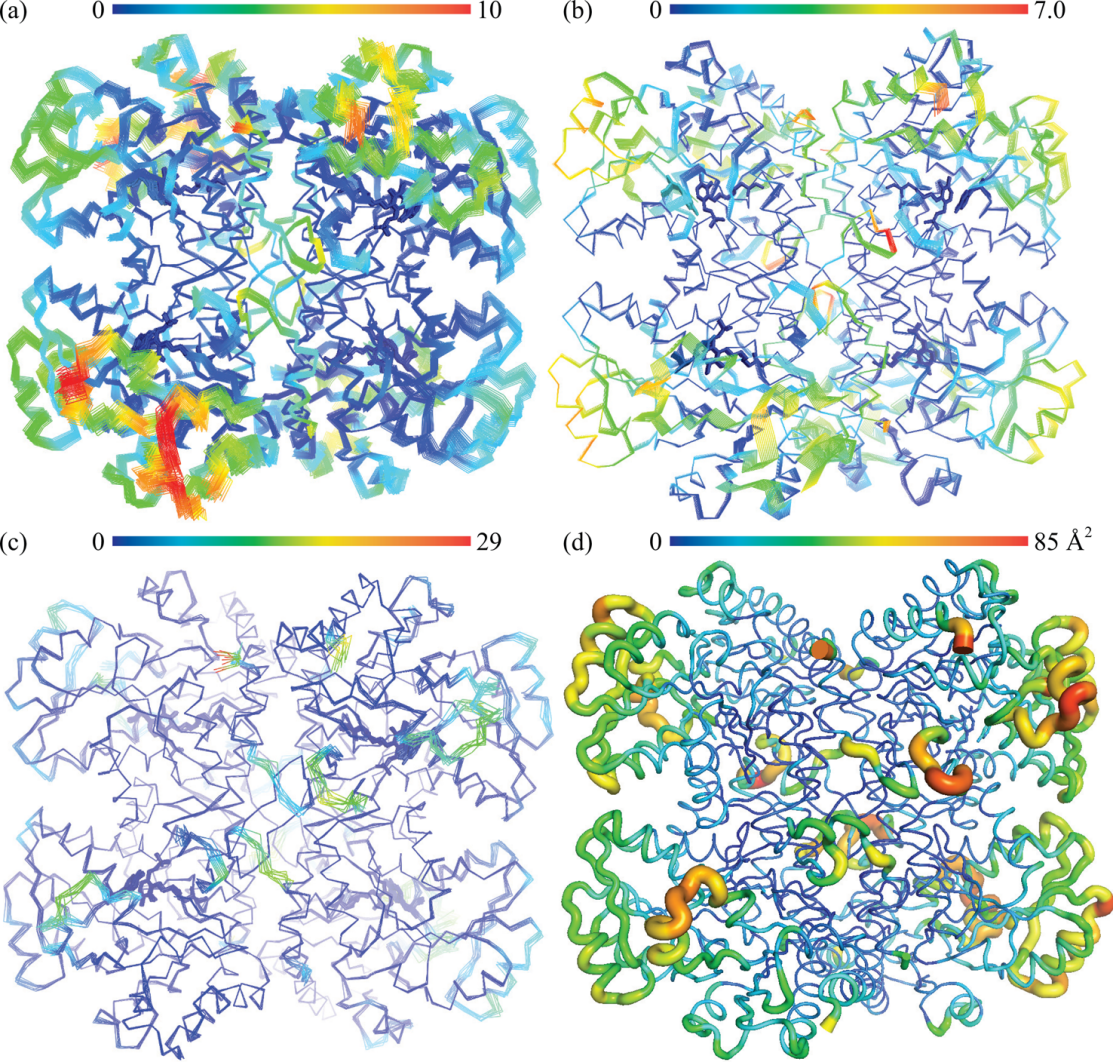
Dynamics modeling and analysis of CalE6. (a) Combination of all top 5 low frequency normal modes simulated by ElNemo server^7^ and colored based on scaled variational distance (x100) using Mobi server.^50^ (b) The top slowest normal mode from ElNemo simulation analyzed by Mobi. (c) Crystallographic ensemble refinement models analyzed by Mobi (PDB entry 4XQ2). (d) Single model crystal structure shown as B-factor putty using PyMOL preset.^46^ B factors of C*α* atoms are represented by both the color spectrum and scaled thickness of the ribbon. All analyses show similar mobility distribution, with normal mode analysis (a, b) showing additional C-terminal region dynamics attributable to crystal contact. The spectrum bar indicates relative mobility from low (blue) to high (red) and covers the range of minimum to maximum values of the corresponding parameters.

**FIG. 5.**
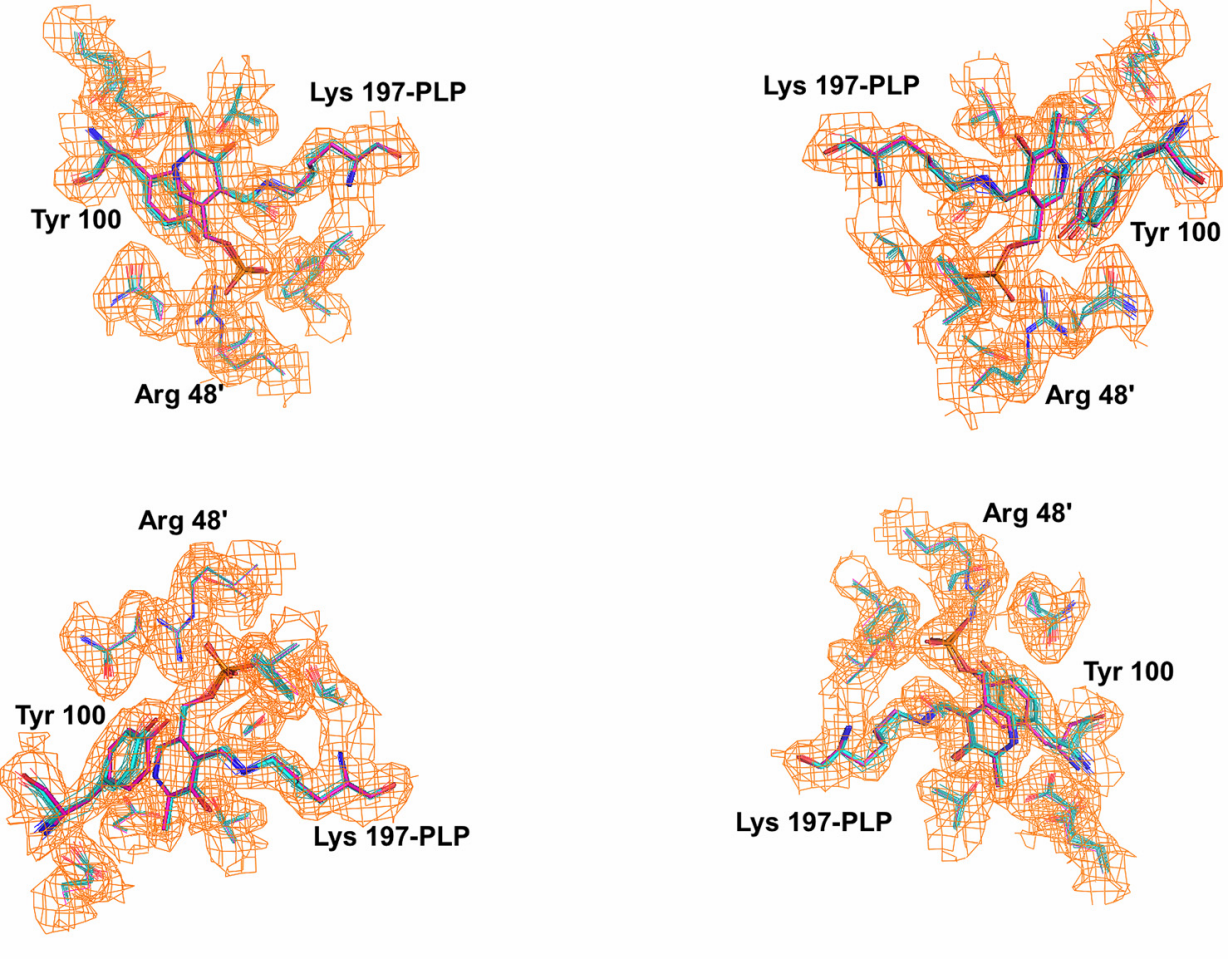
Analysis of ensemble refinement models with reference to two different conformations of CalE6. Structures of the sulfate bound and MES bound forms were aligned using PyMOL^46^ based on the tetrameric form (chains A–D). All four active site residues were shown, with Lys 197-PLP and Tyr 100 highlighted as sticks and the other side chains as lines. Structures were differently colored based on carbon atoms, with ensemble models (teal), MES bound form (cyan), and sulfate bound form (magenta). Non-covalent ligands were omitted. 2Fo-Fc density map was shown for PLP and active site residues at 1.0 σ level.

We next examined dynamic properties of the CalE6 through two orthogonal approaches, normal mode analysis, and crystallographic ensemble refinement. The methods revealed similar mobility distribution as analyzed with Mobi Server^50^ based on scaled RMSD of atomic position in the ensemble models and with B-factor based mobility distributions of the single model crystal structure (Figures 4(a)–4(d)). The N-terminal extended loop for tetrameric assembly and PLP phosphate binding of CalE6 consistently showed relatively high mobility from all the analyses above, which agrees well with the conformational change of the corresponding N-terminal region from disordered to structured upon PLP recruitment into human cystathionine *γ*-lyase (hCSE) (PDB entries 2NMP and 3ELP, 41% sequence identity with CalE6).^59^ A brief survey of crystallographic B-factor distribution of homologs of CalE6 available in PDB (sequence identity 27–45%) suggests nontrivial conservation of intrinsic mobility of the same N-terminal region that links PLP binding to tetramer assembly (data not shown), which is consistent with experimental observation by Sun *et al*. that apo-hCSE exists as a weaker tetramer compared with PLP·hCSE complex in solution.^59^ Elastic network model analysis suggests a similar mobility distribution of the slowest normal mode of CalE6 to the combined ensemble of all top 5 slow modes, both showing additional spatially clustered mobile elements near the C-terminus that are absent in the crystallographic ensemble models (Figures 4(a)–4(c)). This discrepancy between local mobility from normal mode analysis and crystallographic modeling can likely be explained by crystal packing effect where the otherwise mobile elements aforementioned were restricted by interactions between neighbor molecules in the crystal lattice (data not shown). While normal mode analysis simulates overall protein mobility at the backbone level, experimentally based ensemble refinement proves to be useful to extract both major and minor conformations at the side chain level. Ensemble refinement was able to identify the different rotamers of Tyr 100 of CalE6 covering the conformational space as observed in the static crystal structures of different ligand complexes (MES vs. sulfate) (Figure 5). As expected, the pyridine ring of PLP is restricted to a limited conformational space by the internal aldimine linkage with Lys 197 that is distinct from the conformer observed for external aldimine intermediates (Figure 5). This, in essence, reflects the presence of a minor population of alternative conformers per residue in 100 K cryogenic crystal conditions. In agreement, there is clear variation in side-chain mobility for different residues in the vicinity of PLP cofactor and the active site, like the flexible Tyr 100 vs. barely mobile Arg 48 (Figure 5).

## IV. CONCLUSION

We demonstrated through comparative structural analysis of CalE6 and its homologs that the conserved tyrosine residue stacking with PLP is subject to ligand-induced rotation. The same type of concerted motion of PLP and tyrosine pair of PLP-dependent enzymes involved in Cys/Met metabolism is found in multiple external aldimine intermediate structures with respect to their native states,^51–56^ emphasizing a possible dynamic role of this functional side chain during catalysis. The overall dynamics of CalE6, revealed consistently through elastic network model analysis and ensemble refinement, resembles the conformational flexibility of human cystathionine *γ*-lyase required for holoenzyme maturation, which involves both structuring of the otherwise disordered N-terminal loop responsible for PLP phosphate binding and the swing motion of the active site loop to bring the conserved tyrosine closer to PLP for stacking with the pyridine ring.^59^ In summary, the intrinsic dynamics of Tyr 100 of CalE6 can be possibly understood in the context of PLP enzyme function at two levels—catalytic role as an indirect electrostatic modulator or direct proton donor and structural role contributing to cofactor affinity during holoenzyme maturation. Future work of direct observation of these transient dynamic events via pulse-triggered time-resolved experiments can further elucidate the sequence and time scale of individual steps and their functional relevance.

## Figures and Tables

**TABLE I.** Statistics for X-ray data collection and structural refinement of CalE6. (Values in parenthesis are for the highest resolution shell.)

Statistic	Single model refinement	Ensemble refinement
Protein Data Bank ID code	4Q31	4XQ2
Spacegroup	I222	
Cell dimensions		
*a, b, c* (Å)	146.9, 147.0, 349.9	
*α, β, γ* (deg)	90.0, 90.0, 90.0	
Wavelength (Å)	0.97915	
Resolution of data collection (Å)	34.0–2.10 (2.14–2.10)	
No. of unique reflections	218967 (21668)	
Completeness % (Å)	99.9 (99.0)	
Redundancy	7.4 (7.3)	
*R*_sym_^a^	0.14 (0.72)	
I/σ^b^	16.6 (2.9)	
Resolution range in refinement (Å)	34.0 – 2.10 (2.12–2.10)	
No. of unique reflections (work/test)	218929/10997	
*R*_cryst_^c^	15.3 (19.5)	14.5 (17.7)
*R*_free_^d^	19.1 (26.2)	18.3 (24.4)
Mean coordinate error^e^ (Å)	0.19	0.14
Rmsd bond length (Å)	0.007	0.009
Rmsd bond angles (deg)	1.07	1.34
Average B value (Å^2^) (overall/protein/waters/ligand)	29.7/29.1/33.5/48.3	25.3/24.8/24.7/71.6
No. of non-hydrogen atoms	24719	243453
No. of protein atoms	22606	226060
No. of waters	1823	14543
No. of ligands and sugars	8 MES, 28 glycerol, 5 formic acid, 12 Cl^−^	80 MES, 280 glycerol, 30 formic acid, 120 Cl^−^

Ramachandran Statistics^f^ (%)	97, 2.5, 0.5	92, 6.3, 1.7

^a^ *R*_sym_ = ∑_*hkl*_ ∑_*i*_ |*I_i_*(*hkl*) − ⟨*I*(*hkl*)⟩|/ ∑_*hkl*_ ∑_*i*_ *I*_*i*_(*hkl*), where *I_i_*(*hkl*) is the intensity of an individual measurement of the symmetry related reflection and ⟨*I*(*hkl*)⟩ is the mean intensity of the symmetry related reflections.

^b^ I/σ is defined as the ratio of averaged value of the intensity to its standard deviation.

^c^ *R*_cryst_ = ∑_*hkl*_ ||*F*_obs_| − |*F*_calc_||/∑_*hkl*_ |*F*_obs_|, where *F*_obs_ and *F*_calc_ are the observed and calculated structure-factor amplitudes.

^d^ *R*_free_ was calculated as *R*_cryst_ using randomly selected small fractions (5%) of the unique reflections that were omitted from the structure refinement.

^e^ Mean coordinate error was calculated based on maximum likelihood.

^f^ Ramachandran statistics indicate the percentage of residues in the most favored, additionally allowed and outlier regions of the Ramachandran diagram as defined by MolProbity.^48^

**TABLE II.** C*α*-C*β*-C*γ*-C*δ*1 dihedral angle values of the conserved tyrosine stacking with PLP in CalE6 and homologs.

Protein name and function	Induced angle (deg)^a^	Native angle (deg)^a^	Difference angle (deg)	PDB entries
	*MES complex*	*Sulfate complex*		
CalE6 methionine *γ*-lyase	50.9 ± 2.4	67.4 ± 0.7	16.5	4Q31, 4U1T^34^
	*External aldimine*	*Internal aldimine*		
*E. histolytica* methionine *γ*-lyase	59.4 ± 2.3	72.8 ± 2.2	13.4	3AEM,^51^ 3ACZ^52^
*X. oryzae* cystathionine *γ*-lyase	49.4 ± 3.4	67.7 ± 2.6	18.3	4IY7,^53^ 4IXZ^53^
*C. freundii* methionine *γ*-lyase	58.1/58.0	66.7	8.6/8.7	4HF8^54^/4OMA,^55^ 2RFV^56^

^a^ The absolute angle values and standard deviations were calculated by averaging for all the monomer copies in the asymmetric unit of each crystal structure. For *C. freundii* methionine *γ*-lyase, a single value without deviation was used because all the tetramer subunits were related by crystallographic symmetry.
